# Author Correction: The recent rapid expansion of multidrug resistant Ural lineage *Mycobacterium tuberculosis* in Moldova

**DOI:** 10.1038/s41467-024-53809-x

**Published:** 2024-10-29

**Authors:** Melanie H. Chitwood, Caroline Colijn, Chongguang Yang, Valeriu Crudu, Nelly Ciobanu, Alexandru Codreanu, Jaehee Kim, Isabel Rancu, Kyu Rhee, Ted Cohen, Benjamin Sobkowiak

**Affiliations:** 1 Department of Epidemiology of Microbial Disease, Yale School of Public Health, New Haven, CT USA; 2 Department of Mathematics, Simon Fraser University, Burnaby, BC Canada; 3 School of Public Health (Shenzhen), Shenzhen Campus of Sun Yat-sen University, Guangzhou University Town Guangdong, Guangdong, PR China; 4 Phthisiopneumology Institute, Strada Constantin Vârnav 13, Chisinau, Republic of Moldova; 5 Department of Computational Biology, Cornell University, Ithaca, NY USA; 6 Department of Medicine, Weill Cornell Medicine, New York, NY USA

**Keywords:** Epidemiology, Phylogeny, Tuberculosis

Correction to: *Nature Communications* 10.1038/s41467-024-47282-9, published online 05 April 2024

The original version of the manuscript contained errors in the phylogenetic analysis, due to missing positions being imputed as the reference nucleotide in the multiple sequence alignment between the Moldovan and Georgian sequences. This has been corrected by re-running the variant calling using GATK ‘GenotypeGVCF’ with all Moldovan and Georgian lineage 4.2 isolates jointly to produce a new multi-sequence alignment for the comparative analysis. Consequently, the following corrections have been made:

The addition of a statement regarding analysis which states “We analyzed the Georgian isolates using the same sequence analysis pipeline as the Moldova strains and performed joint variant calling on the Ural strains from Moldova and Georgia”.

“Interestingly, these nine Georgian strains did not appear on the same genetic background as the Moldovan strains and were paraphyletic with other MDR and non-MDR Georgian isolates, suggesting there was independent acquisition of these SNPs (Supplementary Fig. 3)” has been corrected to “These nine isolates from Georgia were found to be closely related to the Moldovan Ural MDR strains on a phylogeny, suggesting that these highly transmissible strains are also present outside the Republic of Moldova (Supplementary Fig. 3)”.

The original version of the Supplementary Information associated with this Article contained an error in Supplementary Fig. 3 in the placement of arrows indicating shared SNPs. The HTML has been updated to include a corrected version of the Supplementary Information.

A sentence in the Discussion has been corrected:

“Notably, we found that a small number of the Georgian MDR Ural strains shared multiple mutations that were found only in the Moldovan MDR Ural strains in our study. However, these nine MDR Georgian isolates did not cluster with the Moldovan MDR strains in a phylogeny, suggesting there has been convergent evolution at these loci” corrected to “Notably, we found that a small number of the Georgian MDR Ural strains shared multiple mutations that were found in the Moldovan MDR Ural strains in our study and these Georgian isolates appeared to be closely related to Ural MDR-TB isolates from Moldova.”

A statement has been added to the Methods to provide more detail on the analysis pipeline utilised “For the combined analysis of Georgian and Moldovan Ural lineage 4.2 strains, raw sequence data from Ural strains isolated in Georgia10 were obtained from the European Nucleotide Archive (accession numbers PRJEB39561 and PRJEB5058) and mapped to H37Rv as above. Variant calling was re-run using GATK ‘GenotypeGVCF’ with all Moldovan and Georgian lineage 4.2 isolates jointly to produce a new multi-sequence alignment for the comparative analysis shown in Supplementary Fig. 3.”

These have been corrected in both the PDF and HTML versions of the article.

## Supplementary information

Revised Supplementary Information

