# Supplementary material for: The recent rapid expansion of multidrug resistant Ural lineage Mycobacterium tuberculosis in Moldova
Source: Nat Commun. 2024 Apr 5;15:2962. doi: 10.1038/s41467-024-47282-9 (PMC10997638; doi:10.1038/s41467-024-47282-9)
Supplement: Supplementary file 1 — Supplementary Information [file 41467_2024_47282_MOESM1_ESM.pdf]

Supplementary Table 1 Comparison between DST and WGS drug resistance prediction

	Beijing_A (N=140)	Beijing_B (N=80)	Beijing_C (N=224)	Beijing_D (N=32)	Beijing_E (N=153)	Beijing_F (N=80)	Ural_A (N=219)	Ural_B (N=92)	Ural_C (N=20)	Overall (N=1040)
**Rifampin**										
Agree	136 (97.1%)	75 (93.8%)	217 (96.9%)	32 (100%)	147 (96.1%)	77 (96.3%)	216 (98.6%)	91 (98.9%)	20 (100%)	1011 (97.2%)
Disagree	4 (2.9%)	5 (6.3%)	7 (3.1%)	0 (0%)	6 (3.9%)	3 (3.8%)	3 (1.4%)	1 (1.1%)	0 (0%)	29 (2.8%)

	Beijing_A (N=135)	Beijing_B (N=76)	Beijing_C (N=214)	Beijing_D (N=30)	Beijing_E (N=141)	Beijing_F (N=78)	Ural_A (N=203)	Ural_B (N=87)	Ural_C (N=20)	Overall (N=984)
**Isoniazid**										
Agree	132 (97.8%)	74 (97.4%)	206 (96.3%)	30 (100%)	136 (96.5%)	76 (97.4%)	202 (99.5%)	86 (98.9%)	19 (95.0%)	961 (97.7%)
Disagree	3 (2.2%)	2 (2.6%)	8 (3.7%)	0 (0%)	5 (3.5%)	2 (2.6%)	1 (0.5%)	1 (1.1%)	1 (5.0%)	23 (2.3%)

N denotes the number of tests performed per clade

Supplementary Table 2 *In situ* drug resistance profiles for MDR and non-MDR strains

	Beijing MDR (N=394)	Beijing Non-MDR (N=410)	Ural MDR (N=256)	Ural Non-MDR (N=164)	Overall (N=1224)
**Rifampin**					
No	0 (0%)	406 (99.0%)	1 (0.4%)*	163 (99.4%)	570 (46.6%)
Yes	394 (100%)	4 (1.0%)	255 (99.6%)	1 (0.6%)	654 (53.4%)
**Isoniazid**					
No	0 (0%)	345 (84.1%)	0 (0%)	127 (77.4%)	472 (38.6%)
Yes	394 (100%)	65 (15.9%)	256 (100%)	37 (22.6%)	752 (61.4%)
**Pyrazinamide**					
No	105 (26.6%)	408 (99.5%)	188 (73.4%)	163 (99.4%)	864 (70.6%)
Yes	289 (73.4%)	2 (0.5%)	68 (26.6%)	1 (0.6%)	360 (29.4%)
**Ethambutol**					
No	40 (10.2%)	385 (93.9%)	17 (6.6%)	160 (97.6%)	602 (49.2%)
Yes	354 (89.8%)	25 (6.1%)	239 (93.4%)	4 (2.4%)	622 (50.8%)
**Streptomycin**					
No	2 (0.5%)	327 (79.8%)	1 (0.4%)	153 (93.3%)	483 (39.5%)
Yes	392 (99.5%)	83 (20.2%)	255 (99.6%)	11 (6.7%)	741 (60.5%)

*low-coverage, likely hetero-resistance at this site

Supplementary Table 3 Results from the genome wide association study

	Beijing		Ural	
	MDR	non-MDR	MDR	non-MDR
rpoB S450L	88.58%	0.73%	91.41%	0%
katG S315T	99.49%	15.65%		
rpsL K43R	84.26%	18.10%		

Supplementary Table 4 Demographic make-up of time-based clades

	Beijing_A (N=159)	Beijing_B (N=87)	Beijing_C (N=252)	Beijing_D (N=36)	Beijing_E (N=163)	Beijing_F (N=87)	Ural_A (N=263)	Ural_B (N=95)	Ural_C (N=21)	Overall (N=1163)
**Sex**										
Male	128 (80.5%)	65 (74.7%)	196 (77.8%)	24 (66.7%)	125 (76.7%)	71 (81.6%)	193 (73.4%)	73 (76.8%)	17 (81.0%)	892 (76.7%)
Female	31 (19.5%)	22 (25.3%)	56 (22.2%)	12 (33.3%)	38 (23.3%)	16 (18.4%)	70 (26.6%)	22 (23.2%)	4 (19.0%)	271 (23.3%)
**Age**										
Mean (SD)	43.3 (12.4)	40.0 (14.1)	42.8 (13.7)	45.0 (12.7)	43.3 (12.4)	43.9 (14.2)	40.7 (12.3)	42.2 (13.3)	42.7 (9.47)	42.4 (13.0)
Median [Min, Max]	42.0 [1.00, 79.0]	37.0 [3.00, 71.0]	41.0 [6.00, 86.0]	45.0 [18.0, 68.0]	44.0 [15.0, 80.0]	44.0 [11.0, 80.0]	39.0 [8.00, 81.0]	41.5 [14.0, 86.0]	46.0 [26.0, 59.0]	41.0 [1.00, 86.0]
Missing	2 (1.3%)	2 (2.3%)	3 (1.2%)	0 (0%)	0 (0%)	2 (2.3%)	6 (2.3%)	1 (1.1%)	0 (0%)	16 (1.4%)
**Homeless**										
No	136 (85.5%)	74 (85.1%)	223 (88.5%)	30 (83.3%)	137 (84.0%)	71 (81.6%)	223 (84.8%)	86 (90.5%)	19 (90.5%)	999 (85.9%)
Yes	20 (12.6%)	9 (10.3%)	22 (8.7%)	6 (16.7%)	18 (11.0%)	8 (9.2%)	30 (11.4%)	6 (6.3%)	2 (9.5%)	121 (10.4%)
Missing	3 (1.9%)	4 (4.6%)	7 (2.8%)	0 (0%)	8 (4.9%)	8 (9.2%)	10 (3.8%)	3 (3.2%)	0 (0%)	43 (3.7%)
**Previously_Incarcerated**										
No	99 (62.3%)	72 (82.8%)	191 (75.8%)	30 (83.3%)	139 (85.3%)	74 (85.1%)	193 (73.4%)	82 (86.3%)	18 (85.7%)	898 (77.2%)
Yes	39 (24.5%)	8 (9.2%)	31 (12.3%)	2 (5.6%)	13 (8.0%)	4 (4.6%)	29 (11.0%)	3 (3.2%)	3 (14.3%)	132 (11.4%)
Missing	21 (13.2%)	7 (8.0%)	30 (11.9%)	4 (11.1%)	11 (6.7%)	9 (10.3%)	41 (15.6%)	10 (10.5%)	0 (0%)	133 (11.4%)

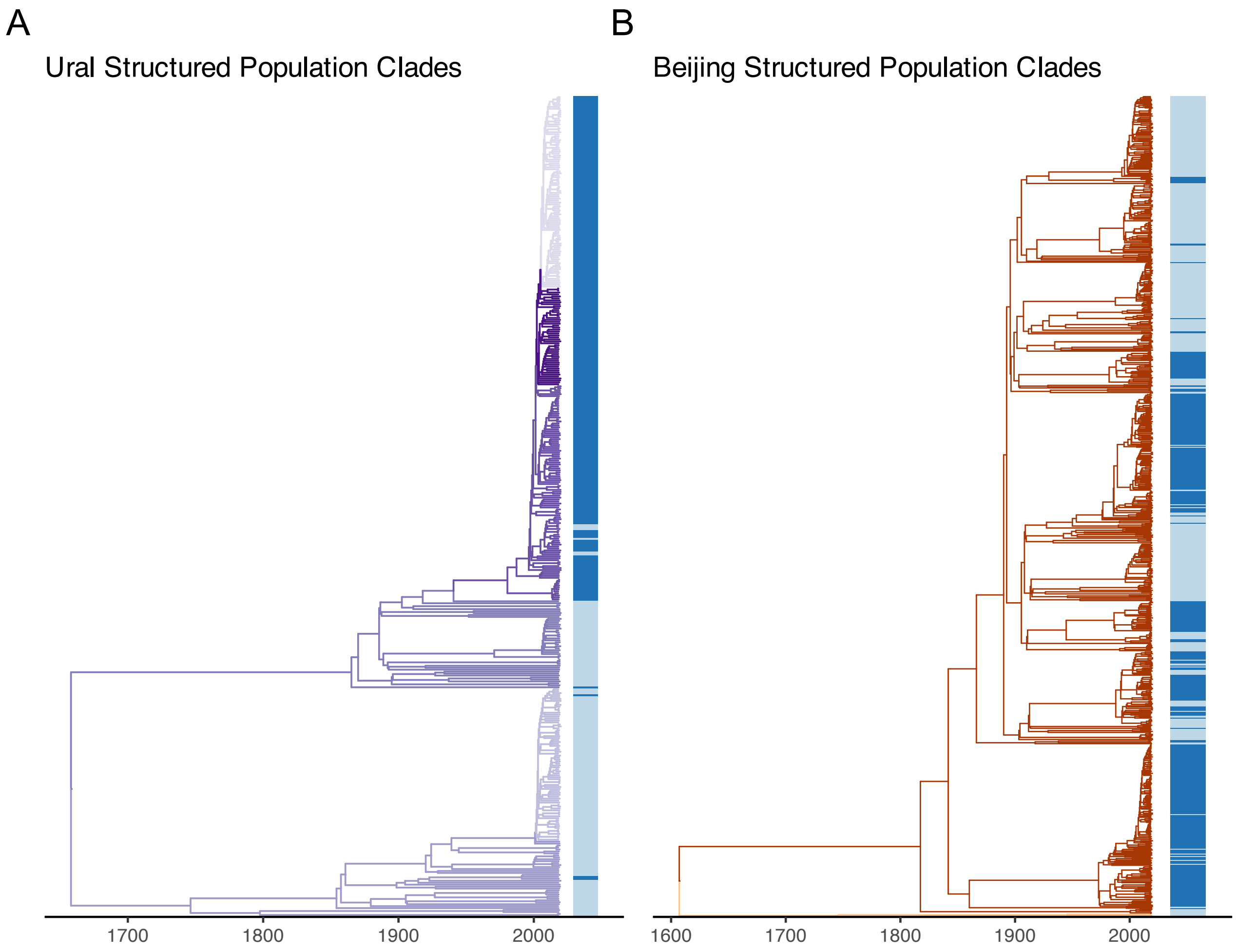

Supplementary figure 1. Timed phylogenetic trees of Ural (A) and Beijing (B) strains used in the study. Branches are colored by structured population clade designations estimated with *TreeStructure*, and the position of MDR (dark blue) and non-MDR (light blue) strains shown in the colored bar.

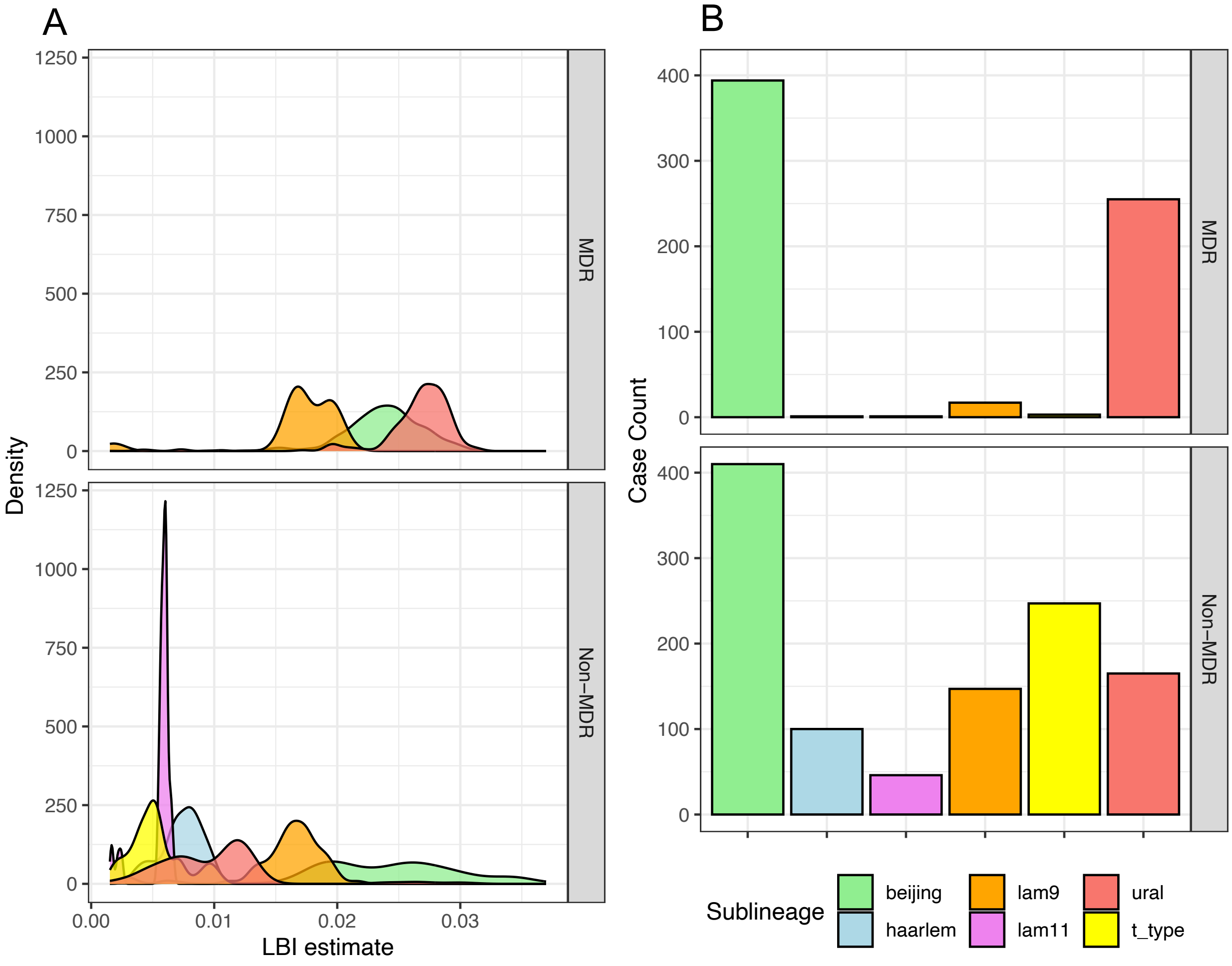

Supplementary figure 2. (A) Local branching index (LBI) estimates for all lineage 2 and lineage 4 sub-lineages present in Moldova during the study period, separated by MDR and non-MDR strain status. (B) Case counts of sequences collected during the study period of each lineage2 and lineage 4 sub-lineage, separated by MDR and non-MDR strain status.

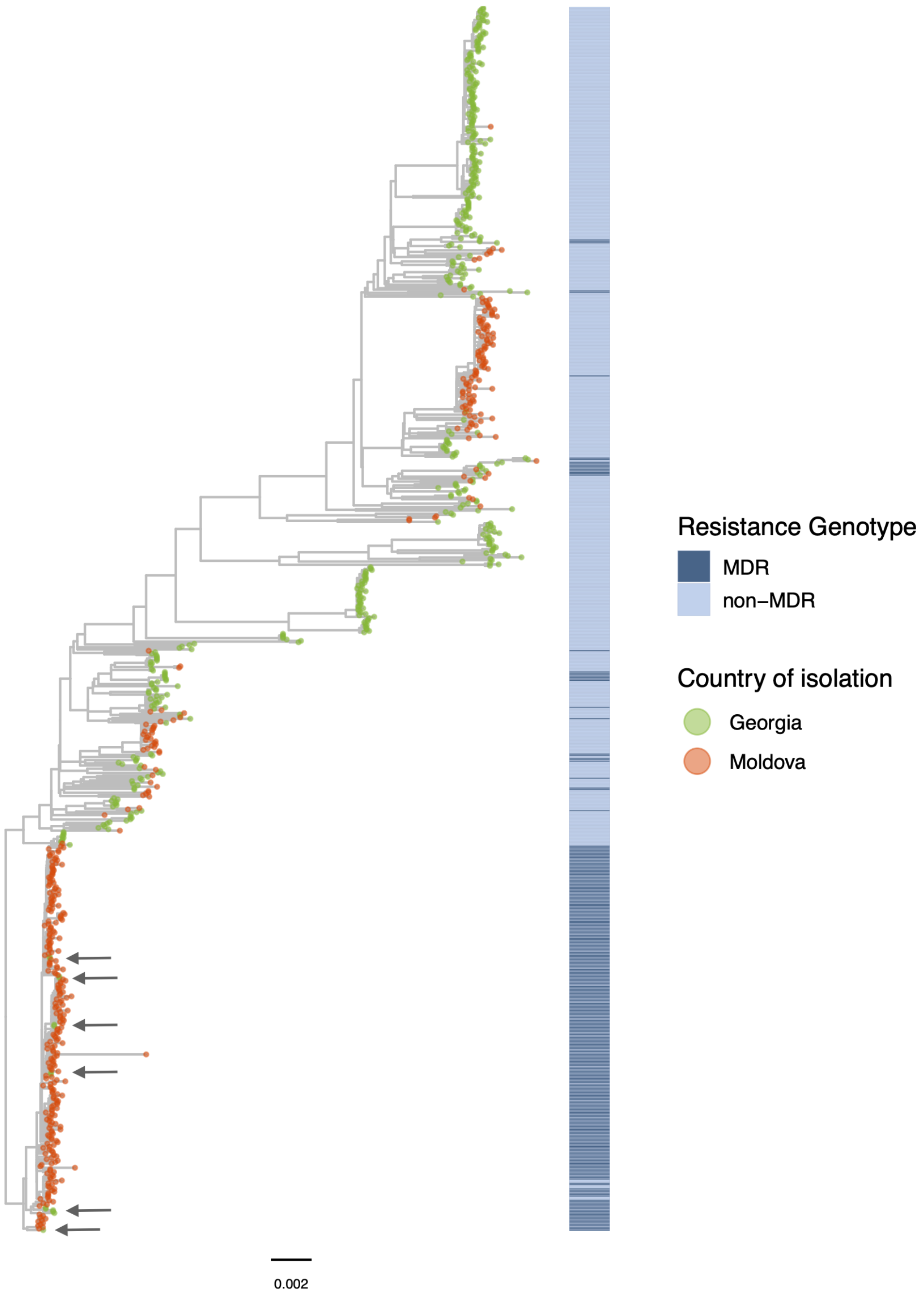

Supplementary figure 3. A maximum likelihood phylogeny of all Ural lineage 4.2 isolates collected in Moldova and Georgia. The tips are annotated by the country of isolation and the resistance genotype (MDR and non-MDR) at the tips illustrated by the colored band. The placement of the nine Georgian MDR strains sharing the SNPs found only in MDR Ural strains from Moldova are indicated by grey arrows.

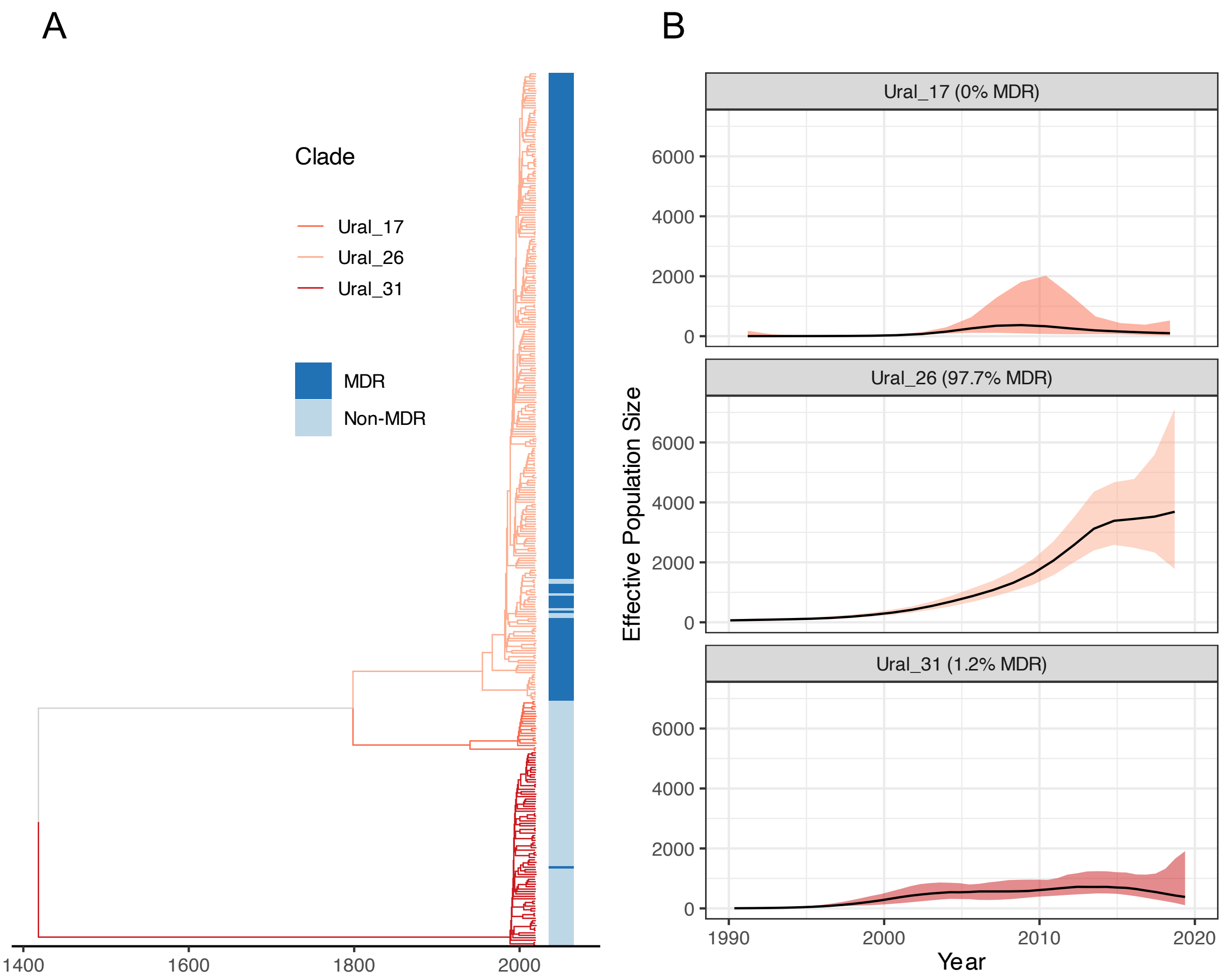

Supplementary figure 4. (A) Timed phylogenetic tree of Ural strains reconstructed using a lower mutation rate of 0.3 SNPs/genome/year, compared to 0.5 SNPs/genome/year in the main analysis, with MDR status and new time-based clade designation shown. (B) Effective population size estimates for each new time-based clade, inferred using *SkyGrowth*, showing the rapid growth of a majority MDR Ural clade (Ural 26), which corresponds to the Ural A clade in the main analysis.

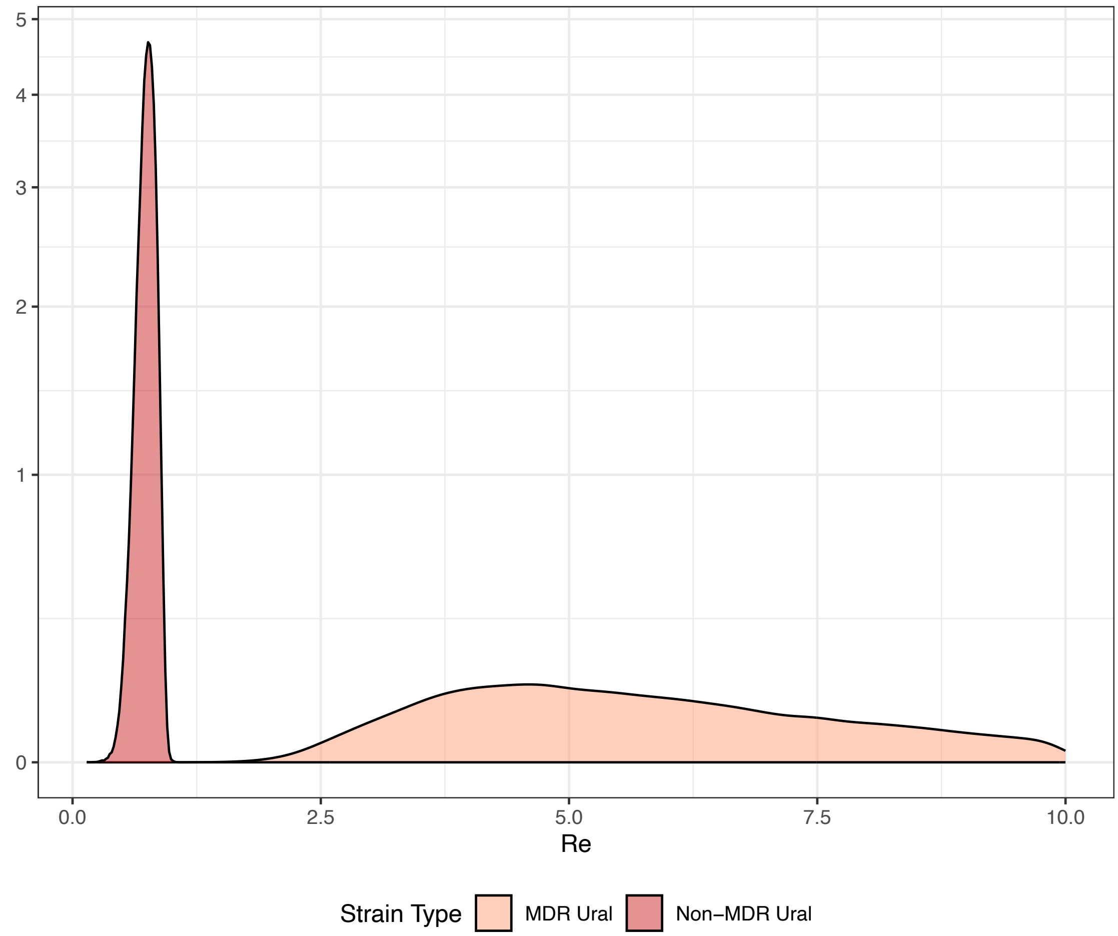

Supplementary figure 5. The effective reproduction number (R_e) of MDR (light red) and non-MDR (dark red) randomly down-sampled Ural strains inferred using a multi-type birth death model in *BEAST2*.
