# Supplementary material for: The recent rapid expansion of multidrug resistant Ural lineage Mycobacterium tuberculosis in Moldova
Source: Nat Commun. 2024 Apr 5;15:2962. doi: 10.1038/s41467-024-47282-9 (PMC10997638; doi:10.1038/s41467-024-47282-9)
Supplement: Supplementary file 3 — Description of Additional Supplementary Files [file 41467_2024_47282_MOESM3_ESM.pdf]

Description of Additional Supplementary files

Supplementary Data 1 : Key mutations and their frequencies in MDR Ural strains from Moldova as compared to non-MDR Ural, MDR Beijing, and non-MDR Beijing from Moldova, and MDR and non-MDR Ural strains from the Republic of Georgia.

Supplementary Data 2 : Name and location of key resistance-conferring mutations on *the M. tuberculosis* genome.
