# Supplementary material for: The recent rapid expansion of multidrug resistant Ural lineage Mycobacterium tuberculosis in Moldova
Source: Nat Commun. 2024 Apr 5;15:2962. doi: 10.1038/s41467-024-47282-9 (PMC10997638; doi:10.1038/s41467-024-47282-9)
Supplement: Supplementary file 6 — Reporting Summary [file 41467_2024_47282_MOESM6_ESM.pdf]

nature portfolio

Corresponding author(s): Ted Cohen

Last updated by author(s): Jan 24, 2024

Reporting Summary

Nature Portfolio wishes to improve the reproducibility of the work that we publish. This form provides structure for consistency and transparency in reporting. For further information on Nature Portfolio policies, see our Editorial Policies and the Editorial Policy Checklist.

Statistics

For all statistical analyses, confirm that the following items are present in the figure legend, table legend, main text, or Methods section.

n/a	Confirmed	
☐	☒	The exact sample size (n) for each experimental group/condition, given as a discrete number and unit of measurement
☒	☐	A statement on whether measurements were taken from distinct samples or whether the same sample was measured repeatedly
☐	☒	The statistical test(s) used AND whether they are one- or two-sided<br>*Only common tests should be described solely by name; describe more complex techniques in the Methods section.*
☒	☐	A description of all covariates tested
☐	☒	A description of any assumptions or corrections, such as tests of normality and adjustment for multiple comparisons
☐	☒	A full description of the statistical parameters including central tendency (e.g. means) or other basic estimates (e.g. regression coefficient) AND variation (e.g. standard deviation) or associated estimates of uncertainty (e.g. confidence intervals)
☐	☒	For null hypothesis testing, the test statistic (e.g. F, t, r) with confidence intervals, effect sizes, degrees of freedom and P value noted<br>*Give P values as exact values whenever suitable.*
☐	☒	For Bayesian analysis, information on the choice of priors and Markov chain Monte Carlo settings
☒	☐	For hierarchical and complex designs, identification of the appropriate level for tests and full reporting of outcomes
☒	☐	Estimates of effect sizes (e.g. Cohen's d, Pearson's r), indicating how they were calculated

Our web collection on statistics for biologists contains articles on many of the points above.

Software and code

Policy information about availability of computer code

Data collection	No data collection software were used.
Data analysis	No previously unreported custom computer code or algorithms were used in this analysis. Raw sequencing data were processed with GATK software and in silico lineage and drug resistance profiling was performed with TB Profiler v.2.8.14. Maximum Likelihood phylogenies were constructed with RAxML-NG v1.2.1. Analyses were performed in R version 4.3.0 with packages BactDating v1.1, TreeImbalance, and Skygrowth. Analyses were also performed in BEAST version 2.7.0 using the bdmm package.

For manuscripts utilizing custom algorithms or software that are central to the research but not yet described in published literature, software must be made available to editors and reviewers. We strongly encourage code deposition in a community repository (e.g. GitHub). See the Nature Portfolio guidelines for submitting code & software for further information.

Data

Policy information about availability of data

All manuscripts must include a data availability statement. This statement should provide the following information, where applicable:

- Accession codes, unique identifiers, or web links for publicly available datasets
- A description of any restrictions on data availability
- For clinical datasets or third party data, please ensure that the statement adheres to our policy

The genomic data from the Republic of Moldova used in this study are available in GenBank under accession code PRJNA736718 (https://www.ncbi.nlm.nih.gov/

bioproject/)13. The genomic data from the Republic of Georgia used in this study are available in European Nucleotide Archive (ENA) at EBI under the accession codes PRJEB39561 and PRJEB505810 (https://www.ebi.ac.uk/ena/browser/home).The H37Rv reference genome used in this study is available from GeneBank under accession code PRJNA57777 (https://www.ncbi.nlm.nih.gov/bioproject/).

Research involving human participants, their data, or biological material

Policy information about studies with human participants or human data. See also policy information about sex, gender (identity/presentation), and sexual orientation and race, ethnicity and racism.

Reporting on sex and gender	We use the term sex to describe the biological attributes of individuals who provided clinical specimens for sequencing. Our findings do not apply to only one sex and sex was not considered in study design. Sex was self-reported int this study. There were 892 males and 271 females in our dataset. We report the breakdown of sex by clade in table S4.
Reporting on race, ethnicity, or other socially relevant groupings	We did not report on race or ethnicity in this study.
Population characteristics	We report on the age, homelessness, and incarceration history of individuals included in the study.
Recruitment	We attempted to recruit all non-incarcerated adults with culture positive TB over a two-year period.
Ethics oversight	Ethical approval was obtained for this study from the Ethics Committee of Research of the Phthisiopneumology Institute in Moldova and the Yale University Human Investigation Committee (Number 2000023071).

Note that full information on the approval of the study protocol must also be provided in the manuscript.

Field-specific reporting

Please select the one below that is the best fit for your research. If you are not sure, read the appropriate sections before making your selection.

☐ Life sciences ☐ Behavioural & social sciences ☒ Ecological, evolutionary & environmental sciences

For a reference copy of the document with all sections, see nature.com/documents/nr-reporting-summary-flat.pdf

Ecological, evolutionary & environmental sciences study design

All studies must disclose on these points even when the disclosure is negative.

Study description	We use previously collected whole genome sequencing data to describe the differences in expansion of specific strains of M. tuberculosis in the Republic of Moldova. This is an observational study and there are no treatment factors, design structure, or experimental units to note.
Research sample	We used WGS data from diagnostic specimens collected from nearly all non-incarcerated adults with culture positive TB over a two-year period. This was a convenience sample based on specimens being processed at the National Laboratory. The sample is representative of non-incarcerated adults with active Tuberculosis in Moldova.
Sampling strategy	We attempted to consent every non-incarcerated adult with culture positive TB. We did not perform a sample size calculation, but rather attempted to have as complete coverage of sequencing as possible. We analyzed only cases belonging to two sublineages -- Beijing 2.2.1 and Ural 4.2.1. No sampling was done; we included all isolates belonging to these two sublineages.
Data collection	In addition to diagnostic specimens collected for sequencing, we collected demographic data (age, sex, homelessness, incarceration history) from routine case report forms and phenotypic drug susceptibility testing results (solid and liquid culture). These data were collected from routine medical and laboratory records. The data were recorded by co-authors NC, VC, and AC.
Timing and spatial scale	The sample covers the entire country of Moldova over a two-year period (January 1 2018 to December 31 2019). We collected samples continuously over the study period; there were no gaps in data collection.
Data exclusions	We excluded 194 polyclonal infections (resulting from concurrent infection with multiple strains).
Reproducibility	We down-sampled our phylogeny to fit a large model in BEAST2. We re-sampled and re-fit the model to ensure that the results were not sensitive to sampling, and we provided all code necessary to reproduce the model results.
Randomization	This is an observational study and we did not randomize individuals to a treatment group.
Blinding	This is an observational study and individuals were not assigned a treatment group; there is nothing to blind.

Did the study involve field work? ☐ Yes ☒ No

Reporting for specific materials, systems and methods

We require information from authors about some types of materials, experimental systems and methods used in many studies. Here, indicate whether each material, system or method listed is relevant to your study. If you are not sure if a list item applies to your research, read the appropriate section before selecting a response.

Materials & experimental systems

n/a	Involved in the study
☒	☐ Antibodies
☒	☐ Eukaryotic cell lines
☒	☐ Palaeontology and archaeology
☒	☐ Animals and other organisms
☒	☐ Clinical data
☒	☐ Dual use research of concern
☒	☐ Plants

Methods

n/a	Involved in the study
☒	☐ ChIP-seq
☒	☐ Flow cytometry
☒	☐ MRI-based neuroimaging

Plants

Seed stocks

Report on the source of all seed stocks or other plant material used. If applicable, state the seed stock centre and catalogue number. If plant specimens were collected from the field, describe the collection location, date and sampling procedures.

Novel plant genotypes

Describe the methods by which all novel plant genotypes were produced. This includes those generated by transgenic approaches, gene editing, chemical/radiation-based mutagenesis and hybridization. For transgenic lines, describe the transformation method, the number of independent lines analyzed and the generation upon which experiments were performed. For gene-edited lines, describe the editor used, the endogenous sequence targeted for editing, the targeting guide RNA sequence (if applicable) and how the editor was applied.

Authentication

Describe any authentication procedures for each seed stock used or novel genotype generated. Describe any experiments used to assess the effect of a mutation and, where applicable, how potential secondary effects (e.g. second site T-DNA insertions, mosiacism, off-target gene editing) were examined.
